# Spatial pattern of foot-and-mouth disease virus serotypes in North Central Nigeria

**DOI:** 10.14202/vetworld.2017.450-456

**Published:** 2017-04-26

**Authors:** Yiltawe Simwal Wungak, Olayinka O. Ishola, Babasola O. Olugasa, David D. Lazarus, David O. Ehizibolo, Hussaini G. Ularamu

**Affiliations:** 1 Department of Veterinary Public Health and Preventive Medicine, Faculty of Veterinary Medicine, University of Ibadan, Ibadan, Nigeria; 2 Division of Virology, National Veterinary Research Institute, Vom, Plateau State, Nigeria

**Keywords:** antibodies, endemic, foot-and-mouth disease, multiple, Nigeria, serotypes, spatial

## Abstract

**Aim:**

This study aimed to determine the foot-and-mouth disease virus (FMDV) serotypes circulating, the prevalence of FMDV serotypes, and the spatial distribution of FMDV among sedentary and pastoral cattle herds in the North-Central Nigeria.

**Materials and Methods:**

A cross-sectional study was undertaken, during which a total of 155 sera that tested positive for foot-and-mouth disease (FMD) 3ABC non-structural protein antibodies were selected and screened for FMD structural protein serotypes, A, O, SAT 1, and SAT 2 using a solid-phase competitive enzyme-linked immunosorbent assay (ELISA). Epithelial tissue specimens were collected during outbreak investigations which were tested for FMD using an antigen capture ELISA for serotype A, O, SAT 1, and SAT 2.

**Results:**

An overall serotype-specific prevalence of 79.35 (95% confidence interval [CI]: 72.4-85.18) was recorded for serotype O, 65.2% (95% CI: 57.41-72.3) for serotype A, 52.9% (95% CI: 45.03-60.67) for SAT 2, and 33.55% (95% CI: 26.45-41.26) for SAT 1. Evidence of exposure to multiple FMDV serotypes showed that 12.26% of the sera samples had antibodies against four serotypes circulating, 30.97% had antibodies against three serotypes circulating, 22.58% had antibodies against two serotypes, and 17% showed exposure to only one serotype. Clinical specimens (epithelial tissue) collected during outbreak investigations showed that serotype O has the highest proportion of 50% with serotype A - 25%; SAT 2 - 20.8%; and SAT 1 - 4.1%.

**Conclusion:**

The study detected diffuse and co-circulation of serotypes A, O, SAT 1, and SAT 2 within the study area, and hence the need for the appropriately matched multivalent vaccine is strongly advocated for FMD control in Nigeria.

## Introduction

Foot-and-mouth disease virus (FMDV) is an infectious disease of cloven-hooved animals including wildlife [1]. It is characterized by high morbidity, vesicle formation and erosion in the mucosa of the mouth, nose, and interdigital space. Foot-and-mouth disease (FMD) is usually associated with devastating economic loss, although mortality is low (about 5%) in adult animals. The economic loss arises from factors such as high calf mortality (50%), decreased calving rate due to infertility and abortion; severe reduction in production of milk and meat because of the characteristic wasting nature of the diseases, loss of draught power resulting from lameness, and loss of access to international market due to trade embargo imposed on importation and exportation of animal meat and animal products from FMD-affected areas [2]. Therefore, to guarantee protection against outbreak situations, an appropriately matched vaccine to the field virus is required [3].

The etiological agent of FMD is classified within the genus *Aphthovirus* in the family *Picornaviridae*. Seven serotypes of FMDV have been identified such as serotypes O, A, C, SAT 1, SAT 2, SAT 3, and Asia 1. It is also known that infection with one serotype does not provoke immune protection to the other serotypes; many strains are identified within serotypes through biochemical and immunological tests [4]. Six of the seven serotypes of FMDV that exist worldwide have been known to circulate in sub-Saharan Africa, namely, A, O, C, SAT 1, SAT 2, and SAT 3 [5]. FMDV in endemic settings across the world has been categorized into six pools; each comprising a different geographic location with different predominant serotypes, and West Africa belongs to pool 5 (O, A, SAT 1, and SAT 2) [6]. Two cycles of FMD occur in sub-Saharan Africa, one where the virus circulates between wildlife and domestic animals and the other where the virus spreads among domestic animals [5]. In some parts of southern and eastern Africa, the cycle between wildlife and domestic animals occurs, while in West Africa, due to the perceived low number of wildlife population, the disease mainly occurs in domestic animals. Four serotypes have been found to be circulating in West Africa (A, O, SAT 1, and SAT 2) [7-10].

FMD remains endemic in Nigeria since the first documented case in 1924 which was attributed to outbreaks in cattle herds caused by serotype O virus [11]. Subsequently, other serotypes (A, SAT 1, and SAT 2) have been identified with transboundary animal movement of trade cattle associated with the outbreaks [7]. Between 2007 and 2009, FMD serotypes A, O, and SAT 2 have been reported to be a major cause of outbreaks in Nigeria [8]. Even though FMD outbreaks are a regular occurrence, laboratory investigation for identification and genotyping of the virus has never been exhaustive and complete, due to poor surveillance system and disease control policy.

The presence of multiple FMD serotypes and the occurrence of subclinical forms of the disease render FMD control very difficult, particularly in pastoral agriculture. This study was designed with the need to identify the FMDV serotypes circulating in the study area, the prevalence of FMDV serotypes, and the spatial distribution of FMDV. The information generated will provide knowledge to researchers, vaccine manufacturers, and policymakers to more efficiently deploy resources to control FMD outbreaks.

## Materials and Methods

### Ethical approval

Ethical approval for this work was granted by the National Veterinary Research Institute, Animal Use and Care Committee (NVRI, AUCC) in December, 2012.

### Study area

**Figure-1:**
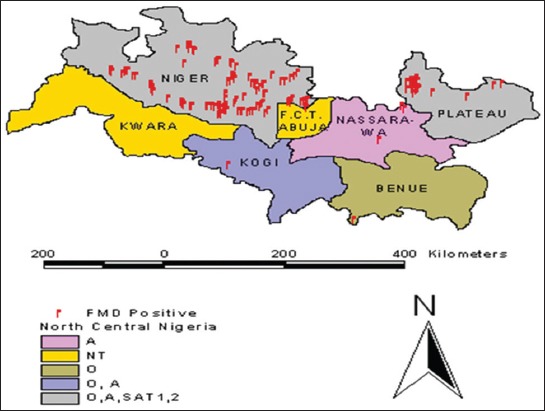
Map of North-Central Nigeria showing foot-and-mouth disease virus serotype distribution within the study areas. NT=Not tested.

The north central zone of Nigeria has a land area of 296,898 km^2^, representing nearly 32% of the country’s total area. It includes Benue, Kogi, Kwara, Niger, Nassarawa, and Plateau states situated between latitudes 6°30’ and 11°20’N (Figure-1). This zone occupies the region of Nigeria and is divided into southern Guinea, derived savannas, and montane subzones. The annual rainfall generally decreases from the South to the North and ranges from 1000 to about 2000 mm per annum with an average of 187-220 rainy days per annum. The zone is dominated by plains, with mountainous areas in the Jos.

The geo-political zone has human population of 20,266,256 and cattle population of 2,363,369 with Kogi (367,754), Kwara (66,905), Nassarawa (88,532), Niger (803,013), Plateau (976,029), and Benue (61,136) [12]. The predominant economic activities are farming and fishing as a result of their fertile nature of land and the presence of rivers Niger and Benue around Kogi, Benue, and part of Niger and some areas involve in mining activities on Jos Plateau Due to the abundance of grassland in the zone, it supports a massive population of livestock and serves as the major cattle trek routes to the eastern and southern parts of the country. The region also shares international boundaries to the west with Benin Republic through Niger and Kwara states [13].

### Study design

A cross-sectional study was undertaken from February 2013 to April 2014; using three-step multistage sampling, 1206 sera were collected from 150 herds in Plateau (589) and Niger (617) states. Tongue epithelial specimens (n=24) from lesions of clinically sick animals were collected purposively between June 2011 and October 2014 from north-central states of Nigeria, namely, Plateau, Kogi, Nassarawa, and Benue. All the cattle herds sampled for this study do not practice FMD vaccination.

### Sample collection for antigen detection

A total of 24 epithelial tissues were purposively collected from reported outbreaks in Plateau, Nassarawa, and Kogi states during 2011-2014. Animals were clinically examined for the presence of FMD lesions on the mouth, teats, nostril, and feet. Epithelial tissue was collected from unruptured or freshly ruptured vesicles and placed in a bottle with virus transport medium composed of the equal amount of glycerol and 0.04 M of phosphate-buffered saline with antibiotics containing penicillin, streptomycin, gentamycin, and amphotericin B within pH 7.2-7.6 [4], and samples were transported to the laboratory on cold chain and stored at −20°C until processed. Field FMD samples were screened using antigen detection enzyme-linked immunosorbent assay (ELISA) for serotypes A, O, SAT 1, and SAT 2 at the National Veterinary Research Institute, Vom, Nigeria.

### Tissue preparation for antigen detection

The tissue samples were prepared as described previously [4]. In brief, the epithelial samples were taken from virus transport medium and were blotted dry on an absorbent paper to reduce the glycerol content, which is toxic for cell cultures and weighed. A suspension was prepared by grinding the sample with sterile sand in a sterile mortar using pestle and with a small volume of tissue culture medium (universal viral transport medium) (Becton, Dickinson and Company, USA). The further quantity of the universal transport medium was added giving a 10% suspension. This was then clarified at 2000 g for 10 min. Clarified sample supernatants were stored at −80°C, for antigen detection and virus isolation. Clarified sample supernatants for virus isolations were filtered through a Millipore filter of 0.22 µm pore size.

### Detection of antibodies against FMDV non-structural proteins (NSPs) ELISA

All the 1206 bovine samples were subjected to FMD screening test; PRIOCHECK FMD-3ABC NSP-ELISA. The PRIOCHECK FMD-3ABC NS protein ELISA kit is designed to detect FMDV-specific antibodies in bovine serum.

The ELISA serology was performed according to the manufacturer’s instructions (PrioCHECKS^®^ Prionics Lelystad, The Netherlands) [14]. Briefly described, 80 µl of the ELISA buffer and 20 µl of the test sera were added to the 3ABC antigen-coated test plates. Negative, weak positive, and strong positive control sera were added to designated wells on each test plate, gently shook, and incubated overnight (18 h) at 22°C. The plates were then emptied and washed 6 times with 200 µl of washing solution, and 100 µl of diluted conjugate was added to all wells. The test plates were sealed and incubated for 60 min at 22°C. The plates were then washed 6 times with 200 µl of the washing solution, and 100 µl of the chromogen (tetra-methyl benzidine) substrate was dispensed to all wells of the plates and incubated for 20 min at 22°C following which 100 μl of stop solution was added to all the wells and mixed gently. Readings were taken on a spectrophotometer Multiskan^®^ ELISA reader (Thermo Scientific, USA) at 450 nm, and the optical density 450 (OD450) values of all samples were expressed as percentage inhibition (PI) relative to the OD450 max using the following formula: PI=100 – (OD450 test sample/OD450 max) × 100. Samples with PI ≥50% were considered positive while those with PI <50% were declared negative. Since the 3-ABC ELISA for FMD was = 100% specific and >99% sensitive, the percentage prevalence was taken as true prevalence [14,15].

### FMDV serotype-specific antibodies

A total of 155 sera tested positive for FMDV NSP by 3ABC ELISA were selected from Niger and Plateau and screened for FMDV serotype-specific FMD antibodies using solid-phase competitive ELISA (SPCE) for antibodies specific to FMDV, serotypes A, O, SAT 1, and SAT 2 to determine the FMDV serotypes circulating in the study area.

### Detection of serotype-specific antibodies against FMDV

The ELISA serology was performed according to the manufacturer’s instructions. A SPCE (IZSLER, Biotechnology Laboratory, Brescia, Italy) was used. The assay is a SPCE using a selected neutralizing anti-FMDV monoclonal antibody (MAb), specific for FMDV serotypes O, A, SAT 1, and SAT 2 to measure antibodies against these serotypes. The test can be applied to measure antibodies in serum or plasma samples of FMDV infected or vaccinated animals of any susceptible species [16].

The FMDV antigen is captured by a serotype-specific MAb, for the serotypes (O, A, SAT 1, and SAT 2) coated to the solid phase with the function of catching Ab. ELISA microplates were supplied pre-coated with FMDV serotype O, A, SAT 1, and SAT 2 antigen captured by the homologous MAb.

The samples were distributed at a single dilution of 1/10, by distributing 45 µl of ELISA diluents buffer and 5 µl of each test serum. In brief, 45 µl of ELISA diluents buffer was added to all the wells excluding negative control wells. A 50 µl volume of negative control serum was added to all the four negative control wells. A 5 µl volume of positive control serum was added to the two positive control wells. A 5 µl volume of test sera was added to all the remaining wells. The plate was gently shaken and incubated for 1 h at room temperature (temperature range: 18-22°C). Without washing, 25 µl of the appropriately diluted horseradish peroxidase conjugate was added to all the wells. The plate was covered and incubated for 1 h at room temperature (temperature range: 18-22°C). After the 1 h incubation, the plate was emptied and 200 µl of washing solution was added and incubated for 3 min at room temperature. The plate was emptied and 3 cycles of washing were repeated leaving the last one for 5 min at room temperature. A 50 µl volume of substrate/chromogen solution was added to all the wells and incubated for 20 min in dark. The reaction was later stopped by the addition of a stop solution and the plates were read on a MultiSkan^®^ spectrophotometer ELISA plate reader (Thermo Scientific, USA) at 450 nm wavelength. Serum end point titer was expressed as the highest dilution producing 50% inhibition, with serum having end point titer ≥50% being classified as positive.

### Detection of FMDV antigen using antigen ELISA

The ELISA serology was performed according to the manufacturer’s instructions. A SPCE from IZSLER, Biotechnology Laboratory (Brescia, Italy) was used.

The assay is a sandwich ELISA that performs with selected combinations of anti-FMDV MAbs, used as coated and conjugated antibodies.

The test can be applied for detection and typing of FMD viruses in homogenates of epithelial and in vesicular fluids. Only in these clinical specimens, the FMD virus usually achieves the concentration required to provide a positive signal in ELISA assays. The kit is designed for detection and typing of FMD viruses of type O, A, SAT 1, and SAT 2. A pan-FMDV test, detecting any isolates of type O, A, C, and Asia1 and, in addition, some of the SATs serotypes, is also included in the kit to complement the specific typing and to detect FMD viruses which might escape binding to the selected type-specific MAb.

Briefly, samples were diluted 1/2 in diluents buffer. A 50 µl volume of each sample was distributed in 12 wells of a row, two replicates for each type-specific catching MAb and for the pan-FMDV-MAb. 50 µl of the diluents buffer was added in all wells of G and H rows. The plate was incubated at room temperature (temperature range: 18-22°C). After the 1 h incubation period, the plate was emptied to remove all the remaining residual fluid. A 200 µl volume of washing solution was added and incubated for 3 min at room temperature (18-22°C). The plate was emptied and the circle of washing was repeated 3 times. After the washing, a 50 µl volume of appropriately diluted conjugate A was added into columns from 1 to 8 and conjugate B from 9 to 12. The plate was incubated for 1 h at room temperature. After the 1 h incubation period, four cycles of washing were repeated as mentioned above leaving the last one for 5 min. After which 50 µl of the substrate-chromogen solution was added to all wells. The plate was covered and left at room temperature (18-22°C) in dark for 20 min. The reaction was later stopped by the addition of a stop solution and the plates were read on a MultiSkan^®^ spectrophotometer ELISA plate reader (Thermo Scientific, USA) at 450 nm wavelength. Results were interpreted according to the protocol criteria for test validity and interpretation based on the manufacturer’s instruction.

### Data analysis

The data were stored in Microsoft Excel^®^ spreadsheet. Descriptive statistics was carried out using Microsoft Excel spreadsheet and proportion was obtained using Open Epi. Version 2.3.1 (Open Source Epidemiological Statistics for Public Health calculator).

Spatially referenced data were presented in ArcGIS 10.1 environment and used for construction of thematic maps of the spatial distribution of FMD serotypes that were identified.

Descriptive and categorical spatial distribution pattern was generated for each FMD serotype identified. Purely spatial scan statistics using Kulldorff [17] method, assuming a Bernoulli distribution pattern, was computed on SatScanVersion 9.1. Significance was set at p<0.05. Clustering analysis was used as a tool to study the spread of diseases in animal populations.

## Results

A total of 155 positive sera for FMDV NSP by 3ABC ELISA were randomly selected from the cattle herds across the geographical zones of Plateau and Niger states and screened against the four FMDV serotypes known to be circulating in Nigeria, i.e. FMDV serotypes A, O, SAT 2, and SAT 1 using SPCE for antibodies specific to serotypes A, O, SAT 2, and SAT 1.

An overall serotype-specific prevalence of 79.35% (123/155, 95% confidence interval [CI]: 72.4-85.18) was recorded for serotype O, 65.2% (95% CI: 57.41-72.3) for serotype A, 52.9% (95% CI: 45.03-60.67) for SAT 2, and 33.55% (95% CI: 26.45-41.26) for SAT 1 (Table-1).

**Table-1:** Prevalence of FMDV serotypes in the study area by serotype-specific Ab-ELISA.

FMDV serotypes	Frequency	Prevalence %	95% confidence limit
A	101	65.20	57.41-72.30
O	123	79.35	72.4-85.18
SAT 1	52	33.55	26.45-41.26
SAT 2	82	52.90	45.03-60.67

FMDV=Foot-and-mouth disease virus, ELISA=Enzyme-linked immunosorbent assay, Ab=Antibody

The results based on states show that 51 sera in Niger states were positive for serotype A, 71 were positive for serotype O, 58 for serotype SAT 2, and 13 were positive for SAT 1, while in Plateau state, results revealed that 50 were positive for serotype A, 49 were positive for serotype O, 82 were positive for SAT 2, and 39 were positive for SAT 1 (Table-2).

**Table-2:** Distribution of FMDV serotypes per state by serotype-specific Ab ELISA.

States	Serotype A %	Serotype 0 %	SAT 1%	SAT 2%
Niger	51	71	13	58
Plateau	50	49	39	24
Total	101	123	52	82

FMDV=Foot-and-mouth disease virus, ELISA=Enzyme-linked immunosorbent assay, Ab=Antibody

Evidence of exposure to multiple FMDV serotypes showed that 12.26% of the sera had evidence of the presence of antibodies against four serotypes circulating, 30.97% had evidence of presence of antibodies against three serotypes circulating, 22.58% had evidence for the presence of antibodies against two serotypes, and 17% show exposure to only one serotype of the virus (Table-3).

**Table-3:** Evidence of exposure to multiple FMDV serotypes by Ab-ELISA.

FMDV serotypes exposure	Proportion	Confidence limit
Four serotypes co-circulating	19 (12.26)	(7.769-18.15)
Three serotypes co-circulating	48 (30.97)	(24.07-38.6)
Two serotypes co-circulating	35 (22.58)	(16.52-29.66)
Single serotype circulating	27 (17)	(12.05-24)

FMDV=Foot-and-mouth disease virus, ELISA=Enzyme-linked immunosorbent assay, Ab=Antibody

Distribution of the circulating FMDV serotypes showed that serotypes A, O, SAT 1, and SAT 2 are circulating in all the geographical zones of Plateau and Niger states in the study area.

The result of the analysis performed by FMDV serotype-specific antigen capture ELISA to determine the presence of FMDV antigen distribution in North-Central Nigeria indicated the co-circulation of four serotypes, namely, FMDV serotypes A, O, SAT 1, and SAT 2 during 2011-2014 (Tables-4 and 5).

**Table-4:** FMD antigen detection ELISA for four serotypes A, O, SAT1, and SAT-2.

Sample ID	Description of sample/sample location	ELISA (IZLER)
KG/OKE/BUKU/5	Bovine epithelium collected 26/06/2011/Kogi state	O
PL/DN/001E	Bovine epithelium collected 20/07/2011/Plateau state	SAT-2
PL/DN/006/E	Bovine epithelium 20/07/2011/Plateau state	SAT-2
NS/DM/008	Bovine probing, 02/08/2011/Nassarawa state	NVD
PL/BK/08185	Bovine epithelium collected 03/11/2011/Plateau state	SAT-2
PL/BK/08196	Bovine epithelium collected 03/11/2011/Plateau state	SAT 2
PL/SH/2012	Bovine epithelium 03/11/2012/Plateau state	-
PL/KA/12M	Bovine epithelium 09/09/2012/Plateau state	O
PL/BLD/02B	Bovine epithelium 06/11/2012/Plateau state	A
PL/BLD/01A	Bovine epithelium 06/11/2012/Plateau state	A
NS/WAM/03	Bovine epithelium 07/11/2012/Nassarawa	A
PL/JS/KA/1	Bovine epithelium collected, 03/01/2014/Plateau state	O
PL/JS/KA 2	Bovine epithelium 03/01/2014/Plateau state	O
PL/JS/KA03	Bovine epithelium collected 03/01/2014/Plateau state	O
PL/KA/4/14	Bovine epithelium collected 14/01/2014/Plateau state	O
Pl/KA/17 B	Bovine epithelium collected 18/01/2014/Plateau state	A
PL/KA/06/04/A-2	Bovine epithelium 14/06/2014/Plateau state	O
PL/KA/06/04/B-2	Bovine epithelium 14/06/2014/Plateau state	O
JS/BI/8/7/14/c	Bovine epithelium collected 18/7/2014/Plateau state	A and O
JS/BI/6/7/14	Bovine epithelium collected 18/7/2014/Plateau state	O
BL/GA/07/14/1	Bovine epithelium collected 20/7/2014/Plateau state	O
BL/GA/07/14/2	Bovine epithelium collected 20/7/2014/Plateau state	O
PL/Js/Vwang/14	Bovine epithelium collected 31-7-14/Plateau state	O
PL/Lang/E	Bovine swab collected 29/11/2012/Plateau state	A, SAT-1, SAT-2

NVD=No virus antigen detected, FMD=Foot-and-mouth disease, ELISA=Enzyme-linked immunosorbent assay

**Table-5:** Distribution of FMDV serotypes circulating in North-Central Nigeria based on antigen detection.

Serotypes	Frequency	Proportion (%)
O	12	50 (30.6-69.4)
A	6	25 (10.8-44.9)
SAT 1	1	4.1 (0.21-18.8)
SAT 2	5	20.8 (8.06-40.3)
Total	24	

FMDV=Foot-and-mouth disease virus

The distribution of FMDV serotypes based on outbreak samples (epithelial tissue) showed that serotype O has the highest proportion (50%) followed by serotype A, 25%; SAT 2, 20.8%; and the lowest as SAT 1, 4.1% (Table-5).

Purely spatial analysis for clusters with high rates using the Bernoulli model revealed clustering of FMD positivity in cattle herds within the coordinates (9.53 N, 8.81) at 3.10 km radius in Jos South local government area of Plateau state; however, the cluster was not statistically significant (p=0.83).

## Discussion

FMD is known to be endemic to most countries in Sub-Saharan Africa, including Nigeria. This study has demonstrated that FMDV remains endemic among pastoral and sedentary husbandry systems within the study areas with multiple serotypes widely distributed. The four FMDV serotypes detected in the course of these studies along with previous reports in Nigeria establish the facts that these are the most prevalent serotypes circulating among cattle in the country [7,9,18,19]. However, to the best of our knowledge, this study has provided the first comprehensive spatial distribution of FMDV serotypes in the North-Central Nigeria, indicating only quadravalent vaccine, including the local isolates containing serotypes A, O, SAT 1, and SAT 2, should be used for vaccination campaign against FMD in Nigeria. From the available information, none of the cattle herd sampled ever practiced FMD vaccination and since routine prophylactic vaccination of cattle is not a common practice in the country, these results tend to present evidence of viral exposure. In this study, analyses of serotype-specific antibodies to FMDV indicate that FMD serotypes O, A, SAT 1, and SAT 2 are widely distributed and co-circulated within the study areas during the period of the study. The wide distribution of the FMDV serotypes in the region could be attributed to unrestricted movement of cattle within the zone, frequent contacts of different herds at watering and feeding points, lack of any meaningful control measure in place, and husbandry management system that is being practiced by the pastoralists. This finding is also in consistent with the previous study conducted between 1960 and 1981, where FMD serotypes A, O, SAT 1, and SAT 2 were detected [20,21]. In addition, between 2007 and 2015, FMD serotypes A, O, and SAT 2 have been reported to cause disease outbreaks among pastoral and sedentary herds in Nigeria [7-9,18,22-24]. These current findings revealed that serotype O is the most prevalent serotype in the region. FMDV serotype O has been known to be the most dominant and most widely distributed serotype. It has the ability to be the most invasive serotype [25]. Depa *et al*. [26] reported that serotype O was most prevalently recorded in most of the FMD outbreaks worldwide. In this study, serotype A was the second in terms of prevalent, followed by SAT 2 and the SAT 1 was the lowest. This is also consistent with a study conducted in Somali Eco-System in Kenya by Chepkwony *et al*. [27] where they reported a higher prevalence of serotype O as compared to the other serotypes.

The evidence of multiple exposures to viruses among herds might be an indication of continuous and silent propagating transmission. This could also be attributed to recent infections with multiple serotypes or reinfection with different serotypes of FMDV over time. This has a great implication for control, as farmers and livestock owners will not have the knowledge of the virus circulating in their herds. This finding is consistent with a study conducted in Uganda where concurrent high Ab titers against serotypes O, SAT 2, SAT 1, and SAT 3 were demonstrated in the same serum samples in cattle herds [28]. Infection or vaccination with one FMD serotype has been known not to confer immune protection against the other serotypes, as such following exposure to a single virus serotype does not provide immunity against the other serotypes of the virus [29]. Another reason is the fact that the presence of antibodies to different serotypes of FMDV is an indication of repeated infection with different serotypes. Alexandersen *et al*. [30] reported that a number of cattle population exposed to FMDV become carriers, in which the animal continues to produce antibodies against the FMDV(s) without showing any clinical sign.

Spatial distribution of the circulating FMDV serotypes showed that serotypes A, O, SAT 1, and SAT 2 are co-circulating in all the six geographical zones of the North-Central Nigeria. This has a great implication in vaccine formulation, as only a multivalent vaccine will be appropriate for use in such situation.

The result of the analysis performed by FMDV serotype-specific antigen capture ELISA revealed the co-circulation of four serotypes, i.e. FMDV serotypes A, O, SAT 1, and SAT 2 during 2011-2014. This is an indication of the fact that FMD is endemic in Nigeria, with the field viruses co-circulating freely in the region; this result is in consonant with a study conducted by Namatovu *et al*. [31] in Uganda where a similar picture was reported. The finding also correlates with detection of FMDV serotype-specific antibodies to serotypes O, A, SAT 1, and SAT 2 in sera samples investigated using serotype-specific antibody detection ELISA in the same North-Central Nigeria as shown above. Factors such as pastoralism, poor movement control, lack of political will for disease control, poor veterinary infrastructures, and lack of adequate workforce might be some of the reasons for the continued transmission of disease among susceptible animals and wildlife population in some parts of the sub-Saharan Africa [32,33]. As reported by several authors, FMD serotype O has been observed to be the predominant serotype from this study.

This result is consistent with a study conducted in Eritrea which showed that serotypes O and A are the most predominant serotypes [34].

Spatial analysis showed that FMD seropositivity and serotypes among cattle herds in the study area were diffuse, which might be as a result of unrestricted movement of cattle within the zone.

## Conclusion

This study detected diffuse circulation and co-circulating of FMDV serotypes A, O, SAT 1, and SAT 2 in cattle herds in North-Central Nigeria; consequently, we recommend movement-controlled measure and the use of multivalent vaccines comprising the four local circulating serotypes as the control option.

## Authors’ Contributions

YSW, DDL, DOE, and HGU carried out sample analysis, edited the manuscript; YSW, OOI, and BOO planned, designed, and conducted data interpretation. All authors read and approved the final manuscript.
